# Implementation of HIV Retesting During Pregnancy and Postpartum in Kenya: A Cross-Sectional Study

**DOI:** 10.9745/GHSP-D-21-00451

**Published:** 2022-02-28

**Authors:** Monalisa Penumetsa, Jillian Neary, Shiza Farid, Peninah Kithao, Barbra A. Richardson, Daniel Matemo, Grace John-Stewart, John Kinuthia, Alison L. Drake

**Affiliations:** a Department of Epidemiology, University of Washington, Seattle, WA, USA.; b Research and Programs, Kenyatta National Hospital, Nairobi, Kenya.; c Department of Global Health, University of Washington, Seattle, WA, USA.; d Department of Biostatistics, University of Washington, Seattle, WA, USA.; e Department of Medicine, University of Washington, Seattle, WA, USA.; f Department of Pediatrics, University of Washington, Seattle, WA, USA.

## Abstract

**Introduction:**

HIV retesting during pregnancy/postpartum can identify incident maternal HIV infection and prevent mother-to-child HIV transmission (MTCT). Guidelines recommend retesting HIV-negative peripartum women, but data on implementation are limited. We conducted a cross-sectional study in Kenya to measure the prevalence of maternal HIV retesting in programs and HIV incidence.

**Methods:**

Programmatic HIV retesting data was abstracted from maternal and child health booklets among women enrolled in a cross-sectional and/or seeking services during pregnancy, delivery, or 9 months postpartum in Kenya between January 2017 and July 2019. Retesting was defined as any HIV test conducted by MTCT programs after the initial antenatal care test or conducted as part of retesting policies at/after delivery for women not tested during pregnancy. Poisson generalized linear regression was used to identify correlates of programmatic retesting among women enrolled at 9 months postpartum.

**Results:**

Among 5,894 women included in the analysis, 3,124 only had data abstracted and 2,770 were enrolled in a cross-sectional study. Overall prevalence of programmatic HIV retesting was higher at 6 weeks (65%) and 9 months postpartum (72%) than in pregnancy (32%), at delivery (23%) and 6 months postpartum (28%) (*P*<.001 for all comparisons). HIV incidence was 0.72/100 person-years (PY) (95% confidence interval (CI)=0.43,1.22) in pregnancy and 0.23/100 PY (95% CI=0.09, 0.62) postpartum (incidence rate ratio: 3.09; 95% CI=0.97, 12.90; *P*=.02).

**Conclusion:**

Maternal retest coverage was high at 6 weeks and 9 months postpartum but low during pregnancy. Strategies to ensure high retesting coverage and detect women with incident maternal HIV infection are needed.

## Abstract (precis)

Strategies are needed to prevent missed opportunities to detect women with incident HIV infection during pregnancy or postpartum and maximize prevention of mother-to-child HIV transmission efforts.

## Teaser

**Key Findings:**

- Despite guidelines to conduct maternal HIV retesting during pregnancy, labor/delivery, and postpartum, we found HIV retesting was inconsistently conducted in Kenyan prevention of mother-to-child HIV transmission programs.
- Programmatic retesting was more frequently implemented at 6 weeks and 9 months postpartum than in the pregnancy or delivery.
- HIV incidence was 3-fold higher during pregnancy than postpartum, suggesting fewer infections acquired postpartum in our study.

**Key Implications:**

- Public health practitioners need to consider strategies to prevent missed opportunities to detect and treat incident HIV infections during and after pregnancy.
- Programs focused on the prevention of mother-to-child HIV transmission should measure maternal retesting and document reasons why retesting does not occur to identify service delivery gaps and improve the implementation of programmatic retesting.
- As mother-to-child HIV transmission rates decline over time with effective interventions, it will be increasingly important to monitor the contributions of maternal HIV incidence to new infant infections to achieve elimination of mother-to-child HIV transmission.

## INTRODUCTION

Globally, 150,000 children became infected with HIV in 2019, the majority of whom acquired HIV through mother-to-child HIV transmission (MTCT).^1^ Early detection and effective treatment of maternal infection are critical to reducing MTCT, making testing a crucial component of prevention of MTCT (PMTCT) interventions. Integration of HIV testing in antenatal care (ANC) and universal antiretroviral therapy (ART) for pregnant and lactating women living with HIV has been a highly effective strategy to reduce MTCT among women with chronic infection. However, newly acquired HIV infections during and after pregnancy may go undetected and untreated.

HIV incidence is high among pregnant and postpartum women, estimated at 2.1/100 person-years (PY) in studies conducted after 2014 in sub-Saharan Africa.^2^ As MTCT risk decreases among women with chronic infection who are effectively treated with ART, the proportion of infant HIV infections attributed to women who acquire HIV infection during pregnancy or postpartum increases.^3^ Incident maternal HIV infections are associated with nearly a 10-fold higher risk of MTCT, in the context of universal ART,^4^ with MTCT rates ranging from 36%–53%.^5^^–^^7^ In the absence of maternal HIV retesting, pregnant and postpartum women who initially test HIV-negative during ANC but acquire HIV later, have undetected infection and miss benefits from PMTCT interventions.

Since 2006, the World Health Organization (WHO) guidelines recommend HIV retesting among HIV-negative pregnant women in the third trimester, at delivery, and/or postpartum.^8^^–^^10^ Kenyan guidelines recommend retesting at the third trimester, delivery (if no retesting in pregnancy), and 6 weeks and thereafter every 6 months until the cessation of breastfeeding.^11^ Yet, data on implementation of maternal HIV retesting throughout pregnancy and postpartum are sparse. In Zambia, retesting during pregnancy was universal among women who had a second ANC visit, but only 67% of all women were retested due to missed visits.^12^ In other studies, in sub-Saharan Africa, HIV retesting during pregnancy ranged from 25%–62%.^13^^–^^15^ Despite guidelines recommending postpartum retesting, comprehensive assessments to measure retesting beyond delivery are lacking.^16^ Data on coverage of maternal retesting can help policy makers adapt guidelines to optimize retesting and elimination of MTCT (EMTCT). We measured the prevalence of HIV retesting in PMTCT programs during pregnancy, delivery, and postpartum; correlates of retesting through 9 months postpartum; and maternal HIV incidence.

Data on coverage of maternal retesting can help policy makers adapt guidelines to optimize retesting and elimination of MTCT.

## METHODS

### Study Design and Participants

#### Cross-Sectional Study

Women were enrolled in a cross-sectional study at 2 health facilities (Ahero County and Bondo Sub-County Hospitals) in the Nyanza region of Kenya. Women seeking ANC or postnatal/infant immunization care services between January 2017 and July 2019 at these facilities were eligible if they were aged 14 years or older and willing to be tested for HIV and provide written informed consent. Additionally, pregnant women were eligible if their gestational age was 28 weeks or more with a history of an HIV-negative test in pregnancy; postpartum women were eligible if they were 6 weeks, 6 months, or 9 months postpartum and either had a documented HIV-negative test result during pregnancy at least 3 months before study enrollment or were not tested for HIV in ANC.

#### Programmatic Abstraction Only Study

As programs began implementing maternal HIV retesting more routinely as the standard of care, making it unnecessary to offer retesting as a research procedure, in January 2019, the study protocol was modified to only abstract programmatic maternal and child health (MCH) data (including HIV testing data retrospectively and at the current visit) from women who had an initial HIV-negative test during pregnancy or were postpartum. We refer to this as the programmatic abstraction only study. Women were eligible for participation in the programmatic abstraction only study if they were aged 14 years or older; if women did not know their age they verified they were at least 14. Women were enrolled only at 6 weeks or 9 months postpartum after giving written informed consent. Study staff only offered retesting in the programmatic abstraction only study if clinics were unable to perform retesting on the day of enrollment (i.e., test kit stock-outs or provider unavailable). In addition to Ahero County Hospital and Bondo Sub-County Hospital, women were enrolled at Siaya County Referral Hospital, Rachuonyo Sub-County Hospital, and Riruta Health Center in Nairobi between January 2019 and July 2019. Following implementation of the programmatic abstraction only study, women were enrolled in the cross-sectional study only if they would not have the opportunity for retesting without study staff offering this procedure.

### Ethics Approval

The Kenyatta National Hospital/University of Nairobi Ethics and Research Committee and the University of Washington Institutional Review Board approved all study procedures.

### Study and Laboratory Procedures

Among women enrolled in the cross-sectional study, a survey was administered by study nurses to collect demographic characteristics, reproductive history, condom use, and male partner characteristics. After survey administration, study staff conducted HIV testing using the Alere Determine HIV-1/2 Ag/Ab Combo test (Abbott Laboratories, Abbott Park, IL), a fourth-generation assay. Women with reactive tests received confirmatory testing using third-generation tests routinely used as the standard of care in Kenya and a tie-breaker test to confirm diagnosis. Participants enrolled from January 2019, were tested with third-generation tests per standard of care. Study staff provided posttest counseling to all women and referred HIV-positive women to MCH clinics for follow-up HIV care and treatment.

Study nurses abstracted age, gestational age, HIV testing, syphilis test results, MCH visit dates, and delivery dates (if applicable) from MCH booklets for women in the cross-sectional and programmatic abstraction only studies.

### Statistical Analysis

Any documented HIV test following initial testing during the pregnancy was classified as a retest; this classification was used to measure the utility of HIV retesting to capture HIV infections in pregnancy that would otherwise be missed without programmatic retesting. In the analysis of programmatic HIV retesting, all maternal retesting conducted by study staff among women enrolled in the cross-sectional study was excluded since retesting was part of eligibility criteria and would overestimate programmatic retesting. Prevalence of third trimester retesting in PMTCT programs was calculated among all women enrolled in the cross-sectional study at delivery or postpartum, and women enrolled in the programmatic abstraction only study during or after the third trimester. Prevalence of HIV retesting at delivery, 6 weeks, 6 months, and 9 months postpartum within PMTCT programs was calculated for women enrolled at or before each of these visits.

HIV retesting prevalence was compared at each time point (third trimester; delivery; 6 weeks, 6 months, and 9 months postpartum) between women enrolled in the programmatic abstraction only and the cross-sectional studies and by year and site. Cofactors for any programmatic HIV retesting among women enrolled in the cross-sectional study at 9 months postpartum were identified using all retesting data available up to but excluding the 9 months postpartum visit. The relationship between age and number of MCH visits (the only nontesting variables abstracted from MCH booklets) and programmatic retesting were examined among women enrolled in the programmatic abstraction only study. The number of ANC and postnatal care (PNC) visits were dichotomized based on the expected number of ANC visits (n=4) and median number of PNC visits among women enrolled at 9 months postpartum in the programmatic abstraction only study. Wilcoxon rank-sum tests were used to compare maternal age and number of MCH visits between women in the programmatic abstraction only and cross-sectional studies. Cofactors for receiving 2 or more retests were identified, which most closely aligns with current Kenyan guidelines^11^ using Poisson generalized linear models with a log-link function; this approach is appropriate when the prevalence of the outcome is high.^17^^,^^18^ Since programmatic HIV retesting procedures may differ by facility (i.e., personnel or guideline implementation), and we detected differences in retesting by site, site clustering was accounted for in the models. Maternal age, education, and marital status were identified as potential confounders *a priori* and variables with *P*<0.1 were included in multivariable models for women in the cross-sectional study. If variables were collinear, variables with the least amount of missing data were included in the multivariable model.

HIV infections detected among pregnant and postpartum women in the cross-sectional and abstraction only studies (including those detected through testing by study nurses) with a prior HIV-negative test during pregnancy (or those with no documentation of an HIV-negative test during pregnancy) were classified as incident infections. Timing of seroconversion was calculated as the midpoint between the last HIV-negative test during pregnancy and first HIV-positive test. Women who tested HIV-positive during pregnancy or less than 72 hours after delivery were classified as having acquired HIV during pregnancy. If the first HIV-positive test was conducted postpartum but the time of seroconversion based on the midpoint calculation was during pregnancy, infections were classified as acquired during pregnancy. Person-time for women with incident infections was calculated as the time between the first HIV-negative test and first HIV-positive test; person-time was stratified by before and after delivery to compare incidence rates during pregnancy vs. postpartum. A sensitivity analysis of incidence rates excluding postpartum women who lacked documentation of an HIV-negative test during pregnancy was conducted. All statistical analyses were performed using STATA v15.1 (College Station, TX).

## RESULTS

### Study Population

Overall, 5,894 women were enrolled; 1,882 (32%) during pregnancy, 130 (2%) at labor and delivery, and 3,882 (66%) postpartum (1,700 (44%) at 6 weeks, 512 (13%) at 6 months and 1,670 (43%) at 9 months) (Table 1 and Supplement Figure 1). The median age was 23 years (interquartile range [IQR]: 21–28). The median number of ANC visits was 3 (IQR: 2–4). Among women enrolled at 9 months postpartum, the median number of PNC visits was 6 (IQR 2–8).

**TABLE 1.** Characteristics of Participants Enrolled in a Cross-Sectional and Programmatic Abstraction Only Study in Kenya, by Enrollment Visit

	**All Women(N=5,894)**		**≥28 Weeks**’ **Gestation(n=1,882)**		**Delivery/Labor(n=130)**		**6 Weeks** **(n=1,700)**		**6 Months(n=512)**		**9 Months(n=1,670)**	
	N	No. (%)	n	No. (%)	n	No. (%)	n	No. (%)	n	No. (%)	n	No. (%)
Age, median (IQR), years	5,889	23 (21–28)	1,881	23 (21–28)	130	23 (20–27)	1,697	23 (20–28)	512	23 (20–27)	1,669	24 (21–28)
Age range, years	5,889		1,881		130		1,697		512		1,669	
<21		1456 (25)		456 (24)		42 (33)		431 (25)		149 (29)		379 (23)
21–30		3705 (63)		1188 (63)		76 (59)		1048 (62)		321 (63)		1072 (64)
≥30		728 (12)		237 (13)		12 (9)		218 (13)		42 (8)		219 (13)
Enrollment site	5,894		1,882		130		1,700		512		1,670	
Ahero		2039 (35)		663(35)		75 (58)		491 (29)		292 (57)		518 (31)
Bondo		2226 (38)		730 (39)		54 (42)		657 (39)		219 (43)		566 (34)
Riruta		503 (9)		50(3)		—		181 (11)		1 (<1)		271 (16)
Siaya		519 (9)		178 (9)		1 (1)		162 (10)		—		178 (11)
Rachuonyo		607 (10)		261 (14)		—		209 (12)		—		137 (8)
Enrollment year^a^	5,894		1,882		130		1,700		512		1,670	
2017		1226 (21)		391 (21)		48 (37)		253 (15)		299 (58)		235 (14)
2018		1237 (21)		537 (29)		81 (63)		220 (13)		212 (41)		187 (11)
2019		3431 (58)		954 (51)		1 (1)		1227 (72)		1 (<1)		1248 (75)
ANC visits documented, median (IQR)^b^	5,854	3 (2–4)	1,842	3 (2–4)	130	2 (2–2)	1,700	3 (2–4)	512	1 (1–2)	1,670	3 (2–4)
PNC visits documented median (IQR)^b^^,^^c^	3,882	2 (1–5)	—	—	—	—	1,700	1 (1–2)	512	2 (1–2)	1,670	6 (2–8)
HIV retests, median (IQR)^d^	5,894	1 (0–2)	1,882	0 (0–1)	130	0 (0–0)	1,700	1 (1–2)	511	1 (1–2)	1,669	2 (1–3)
Incident HIV-infections	5,894	18 (<1)	1,882	8 (<1)	130	1 (1)	1,700	6 (<1)	512	1 (<1)	1,670	2 (<1)

Abbreviations: ANC, antenatal care; IQR, interquartile range; PNC, postnatal care.

a All women enrolled in the years 2017 and 2018 were thorough cross-sectional study only.

b As per maternal and child health booklet documentation. One woman who presented for care at 6 weeks postpartum who was HIV-positive was identified as having an incident infection detected through delivery testing during study screening.

c Among women enrolled at postpartum.

d Including all HIV tests during most recent pregnancy and postpartum, excluding test done as part of study.

Women in the programmatic abstraction only study (n=3,214) were older (median 24, IQR: 21–28 versus median 23, IQR: 20–27; *P*<.001), had more ANC visits (median 4, IQR: 3–5 versus median 2, IQR: 1–2, *P*≤.001) than women in the cross-sectional study (n=2,770) (Table 2 and Supplement Table 1). The number of PNC visits was higher among women in the programmatic abstraction only study than the cross-sectional study (median 7, IQR: 6–8 versus median 2, IQR: 2–3, *P*≤.001), among women enrolled at 9 months postpartum.

**TABLE 2.** Characteristics of Participants Enrolled in a Cross-Sectional Study in Kenya, by Enrollment Visit

	**CROSS-SECTIONAL STUDY (N=2,770)**											
	**All Women(N=2,770)**		**≥28 Weeks’ Gestation(n=928)**		**Delivery/Labor(n=129)**		**6 Weeks(n=540)**		**6 Months(n=512)**		**9 Months(n=661)**	
	n	No. (%)	n	No. (%)	n	No. (%)	n	No. (%)	n	No. (%)	n	No. (%)
**Demographic characteristics**												
Completed secondary education	2,769	1442 (52)	928	530 (57)	129	53 (41)	540	265 (49)	511	243 (48)	661	351 (53)
Completed education (years) median (IQR)	2,766	12 (8–13)	926	12 (8–14)	129	10 (8–12)	539	12 (8–12)	511	11 (8–12)	661	12 (8–13)
Neither parent alive	2,768	357 (13)	926	116 (13)	129	13 (10)	540	60 (11)	512	78 (15)	661	90 (14)
Monthly household income ≥ 10,000 KSH	1,767	695 (39)	596	246 (41)	80	40 (50)	357	133 (37)	345	98 (28)	389	178 (46)
**Relationship characteristics and sexual behavior**												
Married	2,729	1839 (67)	917	665 (73)	127	77 (61)	532	325 (61)	504	340 (67)	649	432 (67)
Currently in a relationship	2,761	1981 (72)	926	705 (76)	129	81 (63)	539	355 (66)	507	364 (72)	660	476 (72)
Relationship duration,^a^ median (IQR), years	1,974	4 (2–7)	701	4 (2–7)	81	4 (2–8)	354	3 (2–7)	362	4 (2–6)	476	4 (2–7)
Polygamous marriage	1,956	141 (7)	696	43 (6)	79	8 (10)	351	33 (9)	360	30 (8)	470	27 (6)
Frequency of sex (last month), median (IQR)	2,438	0 (0–2)	794	0 (0–2)	125	0 (0)	513	0 (0)	449	1 (0–4)	557	2 (0–5)
Any condomless sex (last month)	968	862 (89)	289	262 (91)	21	20 (95)	127	107 (84)	226	202 (89)	305	271 (89)
Age at sexual debut, median (IQR), years	2,485	16 (15–18)	821	16 (15–18)	110	15 (14–18)	489	15 (15–18)	477	16 (15–18)	588	16 (15–18)
Lifetime number of sexual partners, median (IQR)	2,770	2 (1–3)	928	2 (1–3)	129	2 (1–3)	540	2 (1–3)	512	2 (1–3)	661	2 (1–3)
Partner completed secondary education	1,935	1366 (71)	685	500 (73)	78	49 (63)	350	240 (69)	359	251 (70)	463	326 (70)
Partner age difference, median (IQR), years older	1,857	5 (3–7)	671	5 (3–7)	70	5 (4–7)	328	5 (3–7)	340	5 (4–8)	448	5 (3–7)
Partner circumcised	1,918	1338 (70)	684	465 (68)	77	55 (71)	345	245 (71)	351	251 (71)	461	322 (70)
Discussed HIV test with partner prior to test^a^	1,972	1274 (65)	703	448 (64)	80	54 (66)	355	234 (66)	361	241 (67)	473	297 (63)
Partner HIV status^a^	1,981		705		81		355		364		476	
Negative		1309 (66)		446 (63)		51 (63)		228 (64)		259 (71)		325 (68)
Positive		15 (1)		7 (1)		0		0		1 (<1)		7 (1)
Unknown		657 (33)		252 (36)		30 (37)		127 (36)		104 (29)		144 (30)
Partner currently on ART^b^	15	12 (80)	7	4 (57)	0	0 (0)	0	0	1	1 (100)	7	7 (100)
**Reproductive history and clinical history**												
Gravidity, median (IQR)	2,770	1 (1–2)	928	1 (1–2)	129	2 (1–3)	540	1 (1–2)	512	1 (1–2)	661	1 (1–2)
Number of living children^c^^,^ median (IQR)	1,337	2 (1–3)	462	1 (1–2)	66	2 (2–3)	246	2 (2–3)	244	2 (2–3)	319	2 (2–3)
Facility delivery^d^	1,177	1096 (93)	335	303 (90)	58	56 (97)	239	225 (94)	235	216 (91)	310	296 (95)
Ever diagnosed with STI^e^	2,759	31 (1)	926	12 (1)	128	1 (1)	539	4 (1)	512	9 (2)	654	5 (1)
Ever heard of PrEP	1,346	892 (66)	477	343 (72)	41	24 (59)	251	161 (64)	182	117 (64)	395	247 (63)
Ever used PrEP^f^	892	72 (8)	343	27 (8)	24	2 (8)	161	16 (10)	117	8 (7)	247	19 (8)
Incident HIV infections	2,770	9 (<1)	928	5 (1)	0	0 (0)	540	3 (1)	512	1 (<1)	0	0 (0)

Abbreviations: IQR, interquartile range; ART, antiretroviral therapy; PrEP, pre-exposure prophylaxis; STI, sexually transmitted infection.

^a^ Among women with current partners.

^b^ Among women with current partners with HIV.

^c^ Among women with previous pregnancies.

^d^ Among multiparous women including postpartum women who delivered.

**^e^** Self-reported history of STI and rapid plasma reagin test.

^f^ Among women who heard of PrEP.

Among 2,770 women enrolled in the cross-sectional study; 928 (34%) enrolled during the third trimester; 129 (5%) at delivery; and 540 (19%) at 6 weeks, 512 (18%) at 6 months, and 661 (24%) at 9 months postpartum (Table 2). The majority (67%) were married, with a median relationship duration of 4 years (IQR: 2–7). Among 968 women who reported on sexual activity, 89% reported condomless sex the month before enrollment. Overall, 31 (1%) women reported a history of sexually transmitted infections. Most (67%) women with partners said their partner was tested for HIV, 1% (n=15) reported their partners were HIV-positive and 66% (n=1,309) reported their partners were HIV-negative.

### Prevalence of Programmatic Retesting

**FIGURE 1:**
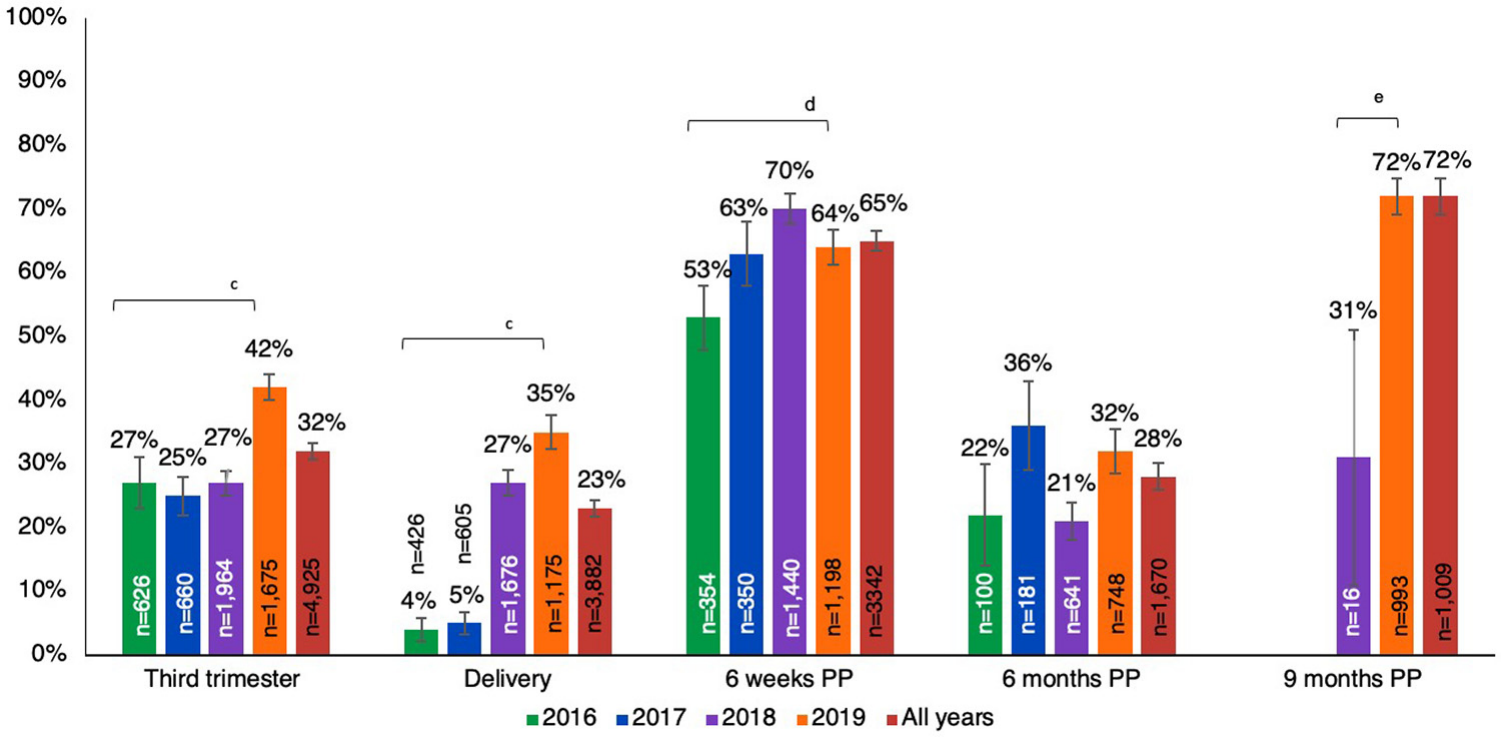
Prevalence of Programmatic Maternal HIV Retesting During Pregnancy and Postpartum in 5 Health Facilities in Kenya by Year^a,b^ Abbreviation: PP, postpartum. ^a^ “All years” is retests between 2016–2019; retests documented in 2015 were omitted due to small sample size (n= 5). From 2016–2017, no women enrolled were eligible for a 9 months postpartum retest. ^b^ The proportion of women retested at each time point was calculated at visits when they enrolled and at all prior time points and retesting conducted as part of the cross-sectional study procedures was omitted; therefore, women can be included in multiple time points. ^c^ Chi-square p-value test for trend to compare across years is <0.001. ^d^ Chi-square p value test for trend to compare across years is <0.01. ^e^ *P*<.001.

**FIGURE 2:**
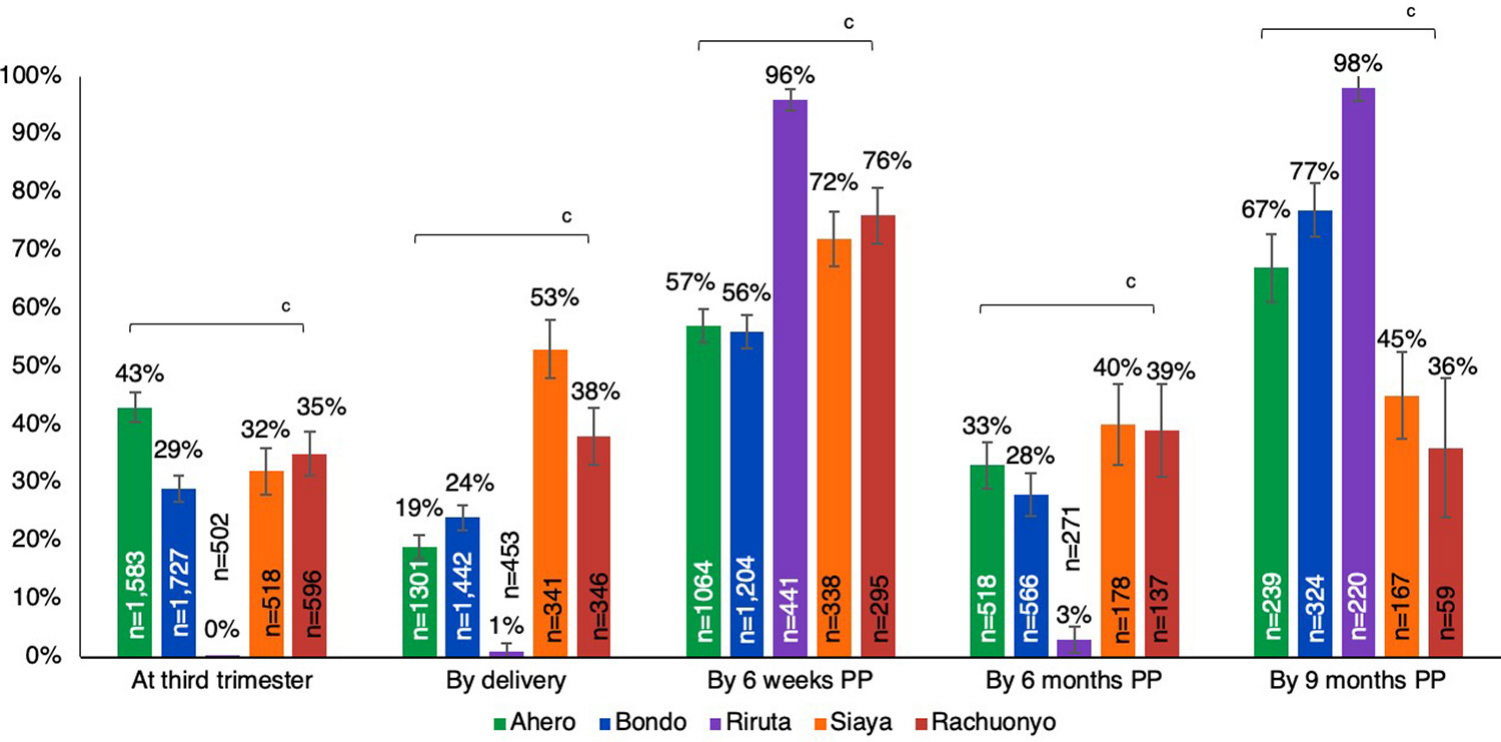
Prevalence of Programmatic Maternal HIV Retesting During Pregnancy and Postpartum in 5 Health Facilities in Kenya by Health Facility^a,b^ Abbreviation: PP, postpartum. ^a^ All women enrolled in the cross-sectional and programmatic abstraction only studies at all sites; retesting conducted as part of the cross-sectional study procedures was omitted. ^b^ The proportion of women retested at each time point was calculated at visits when they enrolled and at all prior time points and retesting conducted as part of the cross-sectional study procedures was omitted; therefore, women can be included in multiple time points. ^c^ *P*<.001.

Among 4,926 women enrolled in either the cross-sectional study or the programmatic abstraction only study, 77% received at least 1 programmatic retest. Only 3% (n=199) did not have documentation of a prior HIV test in pregnancy (n=1 enrolled at delivery, n=22 at 6 weeks, n=61 at 6 months, and n=115 at 9 months postpartum). Among women who lacked documentation of initial testing, 16 women missed multiple opportunities for HIV testing and were tested for the first time during delivery (n=1) or postpartum (n=11 at 6 weeks, n=1 at 6 months, and n=3 at 9 months). Prevalence of programmatic retesting was higher (65%) at 6 weeks and (72%) at 9 months postpartum, than in pregnancy (32%), at delivery (23%), and at 6 months postpartum (28%) (*P*<.001 for all comparisons). Prevalence of programmatic retesting was similar at all time points by study protocol, except for more frequent retesting during pregnancy in the cross-sectional study than the programmatic abstraction only study (38% versus 33%, respectively; *P*=.01). The frequency of programmatic retesting at specific time points increased significantly between 2016 and 2019 during pregnancy (27% to 42%), at delivery (4% to 35%), and 6 weeks postpartum (53% to 64%); no differences in the frequency of retesting were detected at 6 months postpartum. Retesting was significantly higher in 2019 than 2018 (72% versus 31%; *P*<.001) (Figure 1) and differed by site across all years (Figure 2; *P*<.0001).

Programmatic retesting was more frequently implemented at 6 weeks (65%) and 9 months (72%) postpartum than in the third trimester (32%), at delivery (23%), or 6 months postpartum (28%).

We examined completeness of retesting according to Kenyan guidelines among 2,289 women enrolled in the programmatic abstraction only study, with documented visit dates. Overall, 44% of 2,289 women were retested in the third trimester, which increased to 51% when restricted to women enrolled at or after delivery (n=2,128). Coverage of retesting was higher during the postpartum period: 68% of 2,135 women with a PNC visit at 6 weeks retested and 86% of 1,004 women with a PNC visit at either 6 or 9 months retested. However, only 22% of 1,004 women were retested as per Kenyan guidelines (Supplement Figure 2). Among 1,009 women enrolled at 9 months postpartum, 271 (27%) received a retest during pregnancy, 980 (97%) received a retest postpartum, and 261 (26%) received retests both in pregnancy and postpartum.

### Cofactors of Programmatic Retesting

Among 661 women enrolled in the cross-sectional study at 9 months postpartum, 566 (86%) received at least 1 HIV retest: 218 (39%) received 1; 227 (40%) received 2; 97 (17%) received 3; 20 (4%) received 4; and 4 (1%) received 5 retests. While retesting was more common among married than unmarried women (68% versus 32%, respectively; *P*=.01) and women who had more prior pregnancies and less likely among women with a partner who completed secondary education, these associations were not significant in the multivariable model (Supplement Table 2). Being married (*P*=.01), having more previous pregnancies (*P*<.001), and enrolling in 2018 (versus 2017), were associated with retesting at least twice (*P*<.001), while having a partner with unknown or HIV-positive status was associated with a 26% decrease in likelihood of receiving at least 2 retests (*P*<.001) (Table 3). In the adjusted model, only enrollment year remained significant.

**TABLE 3.** Correlates of Participants Receiving At Least 2 HIV Retests by 9 Months Postpartum (N=661), Cross-Sectional Study Only, Kenya

	Received at Least 2 HIV Retests							
	N	No (N=313) No. (%)	N	Yes (N=348) No. (%)	Crude PR^b^ (95% CI)	*P* Value	Adjusted PR^b^ (95% CI)	*P* Value
Sociodemographic characteristics								
Age range, years	313		348				--	--
<21		77 (25)		86 (25)	Ref			
21–30		199 (64)		220 (63)	1.00 (0.85, 1.16)	1.0		
>30		37 (12)		42 (12)	1.01 (0.73, 1.39)	1.0		
Enrollment year	313		348					
2017		117 (35)		118 (34)	Ref		Ref	
2018		86 (37)		101 (29)	1.08 (1.07, 1.08)^c^	<.001	1.07 (1.05, 1.10)	<.001
2019		110 (27)		129 (37)	1.07 (0.60, 1.93)	.8	1.08 (0.62, 1.89)	.8
Completed secondary education	313	179 (57)	348	172 (49)	0.86 (0.74, 1.00)	.06	0.86 (0.75, 1.00)	.05
Neither parent alive	313	36 (12)	348	54 (16)	1.17 (0.92, 1.47)	.2	—	—
Monthly household income ≥10,000KSH	190	90 (47)	199	88 (44)	0.94 (0.72, 1.22)	.6	—	—
Relationship characteristics and sexual behavior								
Married	308	187 (61)	341	245 (72)	1.28 (1.19, 1.38)^c^	<.001	—	—
Polygamous marriage^d^	204	9 (4)	266	18 (7)	1.19 (0.92, 1.55)	.2	—	—
Partner completed secondary education^d^	197	147 (75)	266	179 (67)	0.86 (0.72, 1.04)	0.1	—	—
Partner HIV status unknown/positive^d^	206	75 (36)	270	76 (28)	0.84 (0.79, 0.89)^c^	<.001	—	—
Frequency of condomless sex (last month)	145	131 (90)	160	140 (88)	0.88 (0.63, 1.23)	.5	—	—
Lifetime number of sexual partners	313	98 (31)	348	113 (32)	1.02 (0.88, 1.19)	.7	—	—
Reproductive history								
Gravidity, mean (95% CI)	313	1.77 (1.65, 1.90)	348	2.03 (1.89, 2.18)	1.08 (1.02, 1.14)^c^	.01	—	—
Facility delivery^e^	135	130 (96)	175	166 (95)	0.87 (0.59, 1.29)	.5	—	—

Abbreviations: PR, prevalence ratio; CI, confidence interval.

a Due to collinearity, variables maternal age, marital status, partner HIV status and gravidity were excluded from the multivariate analysis. Variables identified as potential confounders a priori were included in multivariate Poisson GLMs; maternal age, education except marital status.

b Using robust standard errors.

c Statistically significant.

d Among women with current partner.

e Among multiparous women.

Overall, 990 (98%) of 1,009 women enrolled in the programmatic abstraction only study received at least 1 retest, 870 (86%) received at least 2 retests, and 19 (2%) were not retested. Maternal age, number of ANC visits, and number of PNC visits were not associated with receiving at least 1 retest. In an exploratory analysis, women aged 21–30 years were less likely to receive at least 2 retests (prevalence ratio: 0.96, 95% CI=0.94, 0.99; *P*<.01) than younger women, while women who had ≥7 PNC visits (prevalence ratio: 1.17, 95% CI=1.02, 1.34; *P*=.03) were more likely to receive at least 2 retests in a univariate model (Supplement Tables 3 and 4).

### HIV Incidence

Among 5,878 women, we identified 18 (0.3%) with incident maternal HIV infections who were previously HIV-negative; 9 (0.4%) of 2,011 during the third trimester/delivery; and 9 (0.2%) of 3,867 during the postpartum (Table 1) (6 [0.4%] of 1,689 at 6 weeks, 1 [0.2%] of 511 at 6 months, and 2 [0.1%] of 1,667 at 9 months postpartum). After 3,627 person-years of follow-up, the overall incidence rate during pregnancy and postpartum was 0.50/100 PY (95% CI=0.31, 0.79); incidence in pregnancy was 0.72/100 PY; (95% CI=0.43, 1.22), significantly higher than in postpartum (0.23/100 PY; 95% CI=0.09, 0.62; incidence rate ratio (IRR): 3.09; 95% CI=0.97, 12.90, *P*=.02). The overall incidence rate was similar after excluding women with no documented prior negative HIV test during pregnancy. Incidence rates were similar in the cross-sectional and programmatic abstraction only studies (IRR: 1.13; 95% CI=0.40, 3.21, *P*=0.4). All women with incident infections received an initial HIV test during ANC or PNC before testing positive, with the following distribution at the last negative HIV test; 15 (83%) during pregnancy, 1 (6%) at delivery, 1 (6%) at 6 weeks and 1 (6%) at 6 months postpartum.

## DISCUSSION

Despite guidelines to conduct maternal HIV retesting during pregnancy, during labor/delivery, and postpartum, we found HIV retesting was inconsistently conducted in Kenyan PMTCT programs. We found 77% of women were retested during pregnancy or postpartum. A higher proportion of women were retested during pregnancy (32%) than previously reported in other studies conducted in Zambia (25%) and Kenya (10%)^13^^,^^19^ but lower (62%) than a recent study in South Africa.^14^ Few (3%) women had no documentation of HIV testing in pregnancy. Among women with retesting data available through 9 months postpartum, we found retesting was more consistently implemented postpartum: 97% were retested postpartum (26% in both pregnancy and postpartum). Retesting was highest at 6 weeks (65%) and 9 months postpartum (72%). The relatively higher frequency of retesting at 6 weeks postpartum may be due to higher attendance for infant immunizations, prioritization of retesting for HIV by health care providers, provider perceptions of HIV risk during the peripartum period, and/or “catch-up” retesting if retesting in pregnancy and labor/delivery was missed.

Retesting during pregnancy was higher in our study (32%) than in the other study also conducted in Kenya (10%), differences which may be explained by changes in guidelines and gradual roll-out of retesting services.^19^ Guidelines for maternal HIV retesting have changed over time both nationally and globally, with initial recommendations to conduct retesting during pregnancy, and modifications to recommendations to test multiple times during pregnancy and while breastfeeding.^9^^,^^10^^,^^16^ Our study was conducted at a time when scale-up of retesting during pregnancy may have occurred in Kenya, and we did detect an increased frequency of retesting over time, consistent with a study in South Africa.^14^

To determine whether specific groups of women are more likely to be retested, we examined individual- and facility-level correlates of retesting. We found the frequency of retesting was higher among married women, results that are consistent with a prior study in Kenya.^19^ While unmarried women attended fewer ANC visits than married women, the number of ANC visits was not associated with receiving a retest in our study, in contrast to a study conducted in Tanzania.^15^ Women who had several opportunities to be retested in the postpartum were more likely to be retested at least twice in our study. We also found differences in retesting between sites that may be attributed to differences in availability of test kits, clinic volume, or differences in prioritization for retesting by providers by site.

Efforts to improve coverage of retesting will continue to be relevant for EMTCT as the contribution of incident maternal HIV infections to MTCT is expected to increase with successful scale-up of PMTCT interventions for women with chronic HIV infection. Compared to the pre-ART era, risk of MTCT among women with incident versus chronic HIV infections is up to 9-fold higher.^4^ However, effectiveness of retesting for PMTCT will vary based on timing of testing, sexual behavior of peripartum women, and HIV incidence. Recent modeling studies in Kenya suggest that maternal retesting is cost-effective when retesting occurs in late pregnancy with catch-up testing at delivery or 6 weeks postpartum for women without antenatal retesting; however, retesting at 2 or more time points had limited utility and was not cost-effective.^20^

In our study, HIV incidence during pregnancy and postpartum was 0.50/100 PY, which was lower than other recent studies of HIV incidence in Kenya and other parts of sub-Saharan Africa,^2^^,^^21^^–^^24^ and may reflect declines in HIV incidence in Kenya. As scale-up of implementation and frequency of retesting increases, the number of incident maternal HIV infections detected at later time points along the pregnancy-postpartum continuum may decline as women who acquire HIV earlier are identified. Incidence in our study was 3-fold higher during pregnancy than postpartum, which differs from a study noting an increased risk of HIV acquisition per condomless sex act postpartum than during pregnancy^25^ and suggests fewer infections were acquired postpartum in our study. As maternal HIV incidence declines with the implementation of HIV prevention interventions, such as preexposure prophylaxis (PrEP), retesting may become less effective for PMTCT. In a recent, large implementation project in Kenya, more than 20% of HIV-negative pregnant and postpartum women-initiated PrEP, and there were no incident HIV infections among women who used PrEP.^26^ Yet, incident infections were captured in our study in which PrEP was available at clinics and 8% of women reported using PrEP. These results suggest that either higher uptake of PrEP, or more concentrated PrEP use among higher-risk women, will be necessary to reduce the impact of maternal retesting on PMTCT.

One approach to overcome both individual- and facility-level barriers to maternal retesting is HIV self-testing. HIV self-testing decreases provider time associated with routine testing; in busy MCH clinics, this is particularly appealing. Women who have previously received counseling on HIV testing and MTCT may be comfortable with the testing process and prefer a test that uses saliva rather than blood samples. A study in Kenya that offered women the choice of an oral HIV self-test or a routine HIV blood test with a provider found over half of women preferred the oral HIV self-test and cited privacy, ease of use, and less time for testing as reasons for this preference.^27^ Home-based oral HIV self-testing has an additional benefit in that it can be used as a secondary distribution strategy to have male partners tested, and has previously been shown to be highly acceptable to women receiving ANC and postnatal/infant care in sub-Saharan Africa.^28^^–^^31^ While rapid HIV tests that use oral fluid have lower sensitivity than those that use blood samples,^32^ WHO recommends HIV self-testing (including with oral fluid) as a convenient and confidential additional approach for testing, coupled with confirmatory testing with a provider if results are reactive.^33^ Alternative approaches to improve retesting coverage should be tailored to local context and settings but could include maintaining supply chains of test kits to avoid stock-outs and eliminate the need to prioritize recipients of retesting, educating providers to reiterate the need and rationale for retesting, and/or task-shifting to provide sufficient staff time to conduct retesting.

One approach to overcome both individual and facility level barriers to maternal retesting is HIV self-testing.

Our study had several strengths. We abstracted information on maternal HIV retesting during pregnancy, delivery, and throughout the postpartum period from a large number of women to comprehensively assess programmatic retesting. We captured variation in maternal retesting in different sites and over time. We estimated HIV incidence during pregnancy and postpartum and characterized the time points when infections were detected by retesting.

### Limitations

Our study is also subject to some limitations. Our findings may not be generalizable to settings where HIV prevalence is lower or where guidelines for retesting differ. We also did not capture community-, facility- or provider-related factors that may have impacted retesting such as PrEP coverage in the community or clinic, provider skills and trainings, or stock-outs of test kits. In addition, data abstracted from MCH booklets may be incomplete and underreport ANC/PNC visits and HIV testing. Finally, we had limited statistical power to compare incidence rates between pregnancy and postpartum.

## CONCLUSION

In conclusion, maternal HIV retesting is inconsistently implemented in Kenya but has increased over time. Overall, maternal HIV incidence was lower in our study compared to recent studies in other parts of sub-Saharan Africa. Measuring service delivery gaps in offering maternal HIV retesting and impact of retesting programs to detect and treat new maternal HIV infections should be prioritized to monitor progress toward EMTCT.

## Supplementary Material

GHSP-D-21-00451-supplement.pdf

## Notes

**Peer Reviewed**

**First published online:** February 22, 2022.
