# Supplementary material for: Implementation of HIV Retesting During Pregnancy and Postpartum in Kenya: A Cross-Sectional Study
Source: Glob Health Sci Pract. 2022 Feb 28;10(1):e2100451. doi: 10.9745/GHSP-D-21-00451 (PMC8885347; doi:10.9745/GHSP-D-21-00451)
Supplement: GHSP-D-21-00451-supplement.pdf [file GHSP-D-21-00451-supplement.pdf]

Supplement to: Penumetsa M, Neary J, Farid S, et al. Implementation of HIV retesting during pregnancy and postpartum in Kenya: a cross-sectional study. *Glob Health Sci Pract*. 2021;10(1):e2100451. https://doi.org/10.9745/GHSP-D-21-00451

Supplement Figure 1. Total number of women enrolled at each time point by study protocol

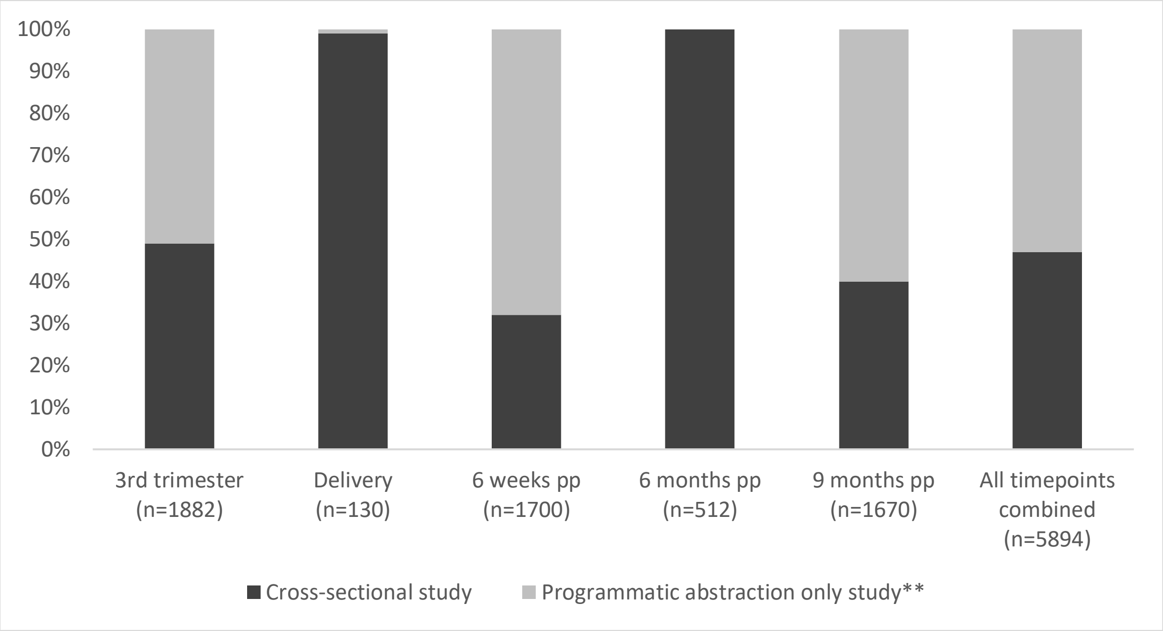

Abbreviation: pp, postpartum. **One woman enrolled into programmatic abstraction only study, who presented for care at 6 weeks postpartum who was HIV positive, was identified as having an incident infection detected through delivery testing during study screening.

Supplement to: Penumetsa M, Neary J, Farid S, et al. Implementation of HIV retesting during pregnancy and postpartum in Kenya: a cross-sectional study. *Glob Health Sci Pract*. 2021;10(1):e2100451. https://doi.org/10.9745/GHSP-D-21-00451

Supplement Figure 2. Prevalence of retesting and complete retesting at each visit, among women in the programmatic abstraction only study

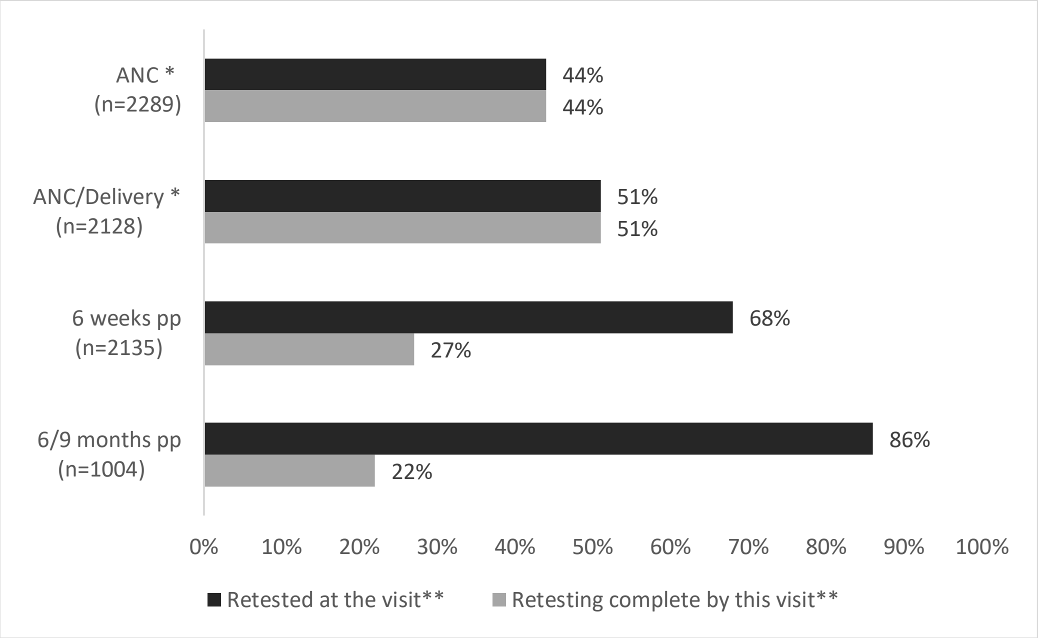

Abbreviations: pp, postpartum; ANC, antenatal care. The proportion of women retesting at each time point was calculated at visits when they enrolled and at all prior time points; therefore, women can be included in multiple time points. * Only one retest is recommended at these time points as per Kenyan guidelines. ** The proportion of women who completed the recommended number of retests up to and including that visit, among women with a recorded visit date. Kenyan guidelines recommend retesting at third trimester/delivery, at 6 weeks postpartum and every 6 months thereafter. Note: Data on facility delivery was not collected and some women may not have delivered at a facility.

Supplement to: Penumetsa M, Neary J, Farid S, et al. Implementation of HIV retesting during pregnancy and postpartum in Kenya: a cross-sectional study. *Glob Health Sci Pract*. 2021;10(1):e2100451. https://doi.org/10.9745/GHSP-D-21-00451

Supplement Table 1. Characteristics of Participants Enrolled in a Programmatic Abstraction Only Study in Kenya, by Enrollment Visit

	All Women (n=3,124)		≥28 Weeks' Gestation (n=954)		Delivery/Labor (n=1)[a]		6 Weeks (n=1,160)		6 Months (n=0)		9 Months (n=1,009)	
	N	No. (%)	n	No. (%)	n	No. (%)	n	No. (%)	n	No. (%)	n	No. (%)
Age, median (IQR), years	3,119	24 (21–28)	953	24 (21–28)	1	31 (31–31)	1,157	24 (21–28)	—	—	1,008	24 (21–28)
Age range, years	3,119		953		1		1,157		—		1,008	
<21		684 (22)		224 (24)		—		245 (21)		—		215 (21)
21–30		1979 (63)		584 (61)		—		742 (64)		—		653 (65)
≥30		456 (15)		145 (15)		1(100)		170 (15)		—		140 (14)
Enrollment site	3,124		954		1		1,160		—		1,009	
Ahero		439 (14)		50(5)		—		169 (15)		—		220 (22)
Bondo		727 (23)		234 (25)		—		254 (22)		—		239 (24)
Riruta		974 (31)		231 (24)		—		419 (36)		—		324 (32)
Siaya		506 (16)		178 (19)		1 (100)		160 (14)		—		167 (17)
Rachuonyo		478 (15)		261 (27)		—		158 (14)		—		59 (6)
ANC visits documented, median (IQR)	3,124	4 (3–5)	954	4 (3–4)	1	0 (0–0)	1,160	4 (3–5)	—	—	1,009	4 (3–5)
PNC visits documented median (IQR)[b]	2,169	2 (2–7)	—	— —	—	— —	1,160	2 (1–2)	—	—	1,009	7 (6–8)
HIV retests, median (IQR)[c]	3,124	1 (1–2)	954	0 (0–1)	1	1 (1–1)	1,160	1 (1–2)	—	—	1,009	2 (2–3)
Incident HIV-infections	3,124	9 (<1)	954	3 (<1)	1	1 (100)	1,160	3 (<1)	—	—	1,009	2 (<1)

Abbreviation: ANC, antenatal care; IQR, interquartile range; PNC, postnatal care.

[a] As per MCH booklet documentation. One woman who presented for care at 6 weeks postpartum who was HIV positive, was identified as having an incident infection detected through delivery testing during study screening.

[b] Among women enrolled at postpartum.

[c] Including all HIV tests during most recent pregnancy and postpartum, excluding test done as part of study.

Supplement to: Penumetsa M, Neary J, Farid S, et al. Implementation of HIV retesting during pregnancy and postpartum in Kenya: a cross-sectional study. *Glob Health Sci Pract*. 2021;10(1):e2100451. https://doi.org/10.9745/GHSP-D-21-00451

Supplement Table 2. Correlates of receiving maternal HIV retesting by 9 months postpartum (N=661), cross-sectional study only

	Received HIV retest							
	N	No [N=95] n (%) or mean (95%CI)	N	Yes [N=566] n (%) or mean (95%CI)	Crude PR[1] (95% CI)	P-value	Adjusted PR[1] (95% CI)	P-value
**Sociodemographic characteristics**								
Age category (years)	95		566					
<21		22 (23)		141 (25)	Ref		Ref	
21-30		62 (65)		357 (63)	0.98 (0.92-1.05)	0.6	0.99 (0.93-1.05)	0.7
>30		11 (12)		68 (12)	1.00 (0.87-1.14)	0.9	1.00 (0.88-1.13)	1.0
Enrollment year	95		566					
2017		44 (46)		191 (34)	Ref			
2018		35 (37)		152 (27)	1.00(0.88-1.13)	1.0	-	-
2019		16 (17)		223 (39)	1.15(0.94-1.40)	0.2	-	-
Completed secondary education	95	52 (55)	566	299 (53)	0.99 (0.95-1.03)	0.6	0.99 (0.96-1.03)	0.7
Neither parent alive	95	10 (11)	566	80 (14)	1.04 (0.97-1.12)	0.2	-	-
Monthly household income ≥10,000KSH	68	31 (46)	321	147 (46)	1.00 (0.98-1.02)	0.9	-	-
**Relationship characteristics and sexual behavior**								
Married	92	51 (55)	557	381 (68)	**1.09 (1.02-1.15)**	**0.01**	**-**	**-**
Polygamous marriage[2]	54	3 (6)	416	24 (6)	1.00 (0.87-1.16)	1.0	-	-
Partner completed secondary education[2]	50	41 (82)	413	285 (69)	**0.94 (0.90-0.97)**	**0.001**	-	-
Partner HIV status unknown/positive [2]	54	21 (39)	422	130 (31)	0.96 (0.91-1.01)	0.1	-	-
Any unprotected sex (last month)	38	33 (87)	267	238 (89)	1.03 (0.88-1.20)	0.7	-	-
Lifetime number of sexual partners	95	31 (33)	566	180 (32)	0.99 (0.93-1.07)	0.9	-	-
**Reproductive history**								
Gravidity*	95	1.64 (1.43-1.85)	566	1.96 (1.85-2.06)	**1.03 (1.01-1.05)**	**0.004**	-	-
Facility delivery (prior pregnancy)[3]	34	31 (91)	276	265 (96)	1.14 (0.97-1.34)	0.1	-	-

Abbreviations: PR, prevalence ratio; CI, confidence interval. [1] Using robust standard errors, [2] Among women with a current partner, [3] Among multiparous women. Variables identified as potential confounders a priori were included in multivariate Poisson GLMs; maternal age, education, and marital status. Due to collinearity, marital status, partner education and gravidity were excluded from the multivariate model. * Per unit change.

Supplement to: Penumetsa M, Neary J, Farid S, et al. Implementation of HIV retesting during pregnancy and postpartum in Kenya: a cross-sectional study. *Glob Health Sci Pract*. 2021;10(1):e2100451. https://doi.org/10.9745/GHSP-D-21-00451

Supplement Table 3. Correlates of receiving any HIV retests by 9 months postpartum (N=1009), programmatic abstraction only study

	Received HIV retest									
		No [N=19]			Yes [N=990]		Crude PR	p-value	Adjusted PR	p- value
	N	n (%) or mean (95%CI)		N	n (%) or mean (95%CI)		(95% CI)		(95% CI)	
**Sociodemographic characteristics**										
Age category (years)	19			989						
<21		4	(21)		211	(21)	Ref		Ref	
21-30		13	(68)		640	(65)	1.00 (0.99-1.01)	0.8	1.00 (0.85-1.16)	1.0
≥30		2	(11)		138	(14)	1.00 (0.97-1.04)	0.8	1.00 (0.81-1.24)	1.0
≥4 ANC visits	19	5	(26)	990	610	(62)	1.03 (0.97-1.09)	0.3	-	-
≥7 PNC visits	19	6	(32)	990	746	(75)	**1.04 (0.99-1.10)**	**0.08**	1.05 (0.90-1.21)	0.6

Abbreviations: PR, prevalence ratio; CI, confidence interval; ANC, antenatal care; PNC, postnatal care. Variable identified as potential confounders a priori were included in multivariate Poisson GLMs; maternal age.

Supplement to: Penumetsa M, Neary J, Farid S, et al. Implementation of HIV retesting during pregnancy and postpartum in Kenya: a cross-sectional study. *Glob Health Sci Pract*. 2021;10(1):e2100451. https://doi.org/10.9745/GHSP-D-21-00451

Supplement Table 4. Correlates of receiving ≥ 2 HIV retests by 9 months postpartum (N=1009), programmatic abstraction only study

	Received ≥2 HIV retests									
		No [N=139]			Yes [N=870]		Crude PR	p-value	Adjusted PR	p- value
	N	n (%) or mean (95%CI)		N	n (%) or mean (95%CI)		(95% CI)		(95% CI)	
**Sociodemographic characteristics**										
Age category (years)	138			870						
<21		24	(17)		191	(22)	Ref		Ref	
21-30		95	(69)		558	(64)	**0.96 (0.94-0.99)**	**<.01**	**0.96 (0.94-0.99)**	**<.01**
≥30		19	(14)		121	(14)	0.97 (0.87-1.09)	0.6	0.97 (0.87-1.09)	0.6
≥4 ANC visits	139	58	(42)	870	557	(64)	1.14 (0.97-1.34)	0.1	-	-
≥7 PNC visits	139	79	(57)	870	673	(77)	**1.17 (1.02-1.34)**	**0.03**	**-**	**-**

Abbreviations: PR, prevalence ratio; CI, confidence interval; ANC, antenatal care; PNC, postnatal care. Variable identified as potential confounders a priori were included in multivariate Poisson GLMs; maternal age. Due to collinearity, variable PNC visits variable was excluded from the multivariate model.
